# Torque control in camouflage treatment of a borderline adult skeletal class III malocclusion with en-masse mandibular distalization using buccal-shelf miniscrews: A case report

**DOI:** 10.1097/MD.0000000000049613

**Published:** 2026-07-03

**Authors:** Viet Anh Nguyen, Thi Minh Anh Ha, Viet Hoang, Anand Marya

**Affiliations:** a School of Dentistry, Hanoi Medical University, Hanoi, Vietnam; b Private Practice, Viet Anh Orthodontic Clinic, Hanoi, Vietnam; c Digital Industrial Design and Manufacturing Research Unit, Faculty of Engineering at Sriracha, Kasetsart University, Sriracha, Chonburi, Thailand; d The City of London Dental School, University of Greater Manchester, Bolton, UK; e Department of Orthodontics, Faculty of Dentistry, University of Puthisastra, Phnom Penh, Cambodia.

**Keywords:** buccal-shelf miniscrews, class III malocclusion, extra-alveolar anchorage, mandibular total-arch distalization, orthodontic camouflage, temporary anchorage devices

## Abstract

**Rationale:**

Adult skeletal class III patients who decline orthognathic surgery may be treated with nonsurgical dentoalveolar camouflage. Extra-alveolar anchorage on the mandibular buccal shelf enables en-masse total-arch distalization and clinically meaningful sagittal correction without relying on patient compliance.

**Patient concerns:**

A 19-year-old male presented with an edge-to-edge anterior relationship and generalized spacing. He was satisfied with his facial appearance and declined surgery.

**Diagnoses:**

The patient was diagnosed with a borderline adult skeletal class III malocclusion characterized by a mildly hypodivergent pattern, concave profile, bilateral angle class III canine and molar relationships, edge-to-edge anterior relationship, anterior spacing, maxillary incisor proclination, and mild mandibular incisor lingual inclination.

**Interventions:**

Fixed self-ligating brackets were used with proactive torque control including lower incisor brackets inversion to express positive torque and 0.019 × 0.025-in stainless-steel archwires with added torque. After extraction of the mandibular third molars, 2 buccal-shelf miniscrews with power arms delivered distalizing forces.

**Outcomes:**

Bilateral class I relationships, space closure, and positive overjet were achieved. Cephalometric changes indicated approximately 4 to 5 mm of mandibular dentoalveolar distalization with controlled tipping and lingual root movement, while the Wits appraisal improved substantially despite slight changes in anteroposterior skeletal measures. Favorable sagittal changes were interpreted cautiously because they may reflect mandibular clockwise rotation and dentoalveolar effects rather than true basal skeletal correction.

**Lessons:**

In carefully selected borderline adult class III patients, buccal-shelf miniscrew–anchored en-masse mandibular distalization can deliver upper-range camouflage when torque- and vertical-control biomechanics are actively maintained. Soft-tissue improvements remain limited compared with orthognathic surgery.

## 1. Introduction

Skeletal class III malocclusion is a relatively uncommon but clinically significant deformity with marked geographic and ethnic variation. A global systematic review reported a pooled prevalence of approximately 5.9% in the permanent dentition (0.7–19.9%), with higher rates in Asian populations.^[1]^ Functionally and psychosocially impactful, class III discrepancies often persist into adulthood and require individualized treatment planning.^[2]^

In adults, management typically hinges on either orthognathic surgery for definitive skeletal correction or orthodontic camouflage, the latter increasingly enabled by temporary anchorage devices (TADs). Extra-alveolar anchorage on the mandibular buccal shelf allows en-masse total-arch distalization and several millimeters of sagittal correction in appropriately selected “borderline” cases, albeit with predictably smaller soft-tissue changes than surgery.^[2,3]^ In fact, Kim et al reported that mandibular incisors retroclined by 5.52° when retracted 2.38 mm through total-arch distalization, underscoring the risk of excessive lingual tipping when attempting large-magnitude distalization in a skeletal class III camouflage case.^[4]^

Despite growing adoption, evidence gaps remain. High-quality controlled studies on the long-term stability of buccal-shelf-anchored total-arch distalization relative to mandibular setback surgery are limited, although emerging matched-cohort data suggest comparable short-term posttreatment stability in borderline cases.^[5]^ Against this background, the objective of this case report is to present a carefully selected borderline adult skeletal class III patient managed nonsurgically with en-masse mandibular total-arch distalization using buccal-shelf miniscrews under strict torque control, to quantify the magnitude of correction and occlusal outcomes, and to contextualize these results within current evidence while noting practical considerations.^[6]^

## 2. Case presentation

### 2.1. Diagnosis and etiology

A 19-year-old male presented to the orthodontic clinic with the chief complaint of an edge-to-edge incisal relationship and spacing of the anterior teeth. He was in good general health with an unremarkable medical history.

**Figure 1.**
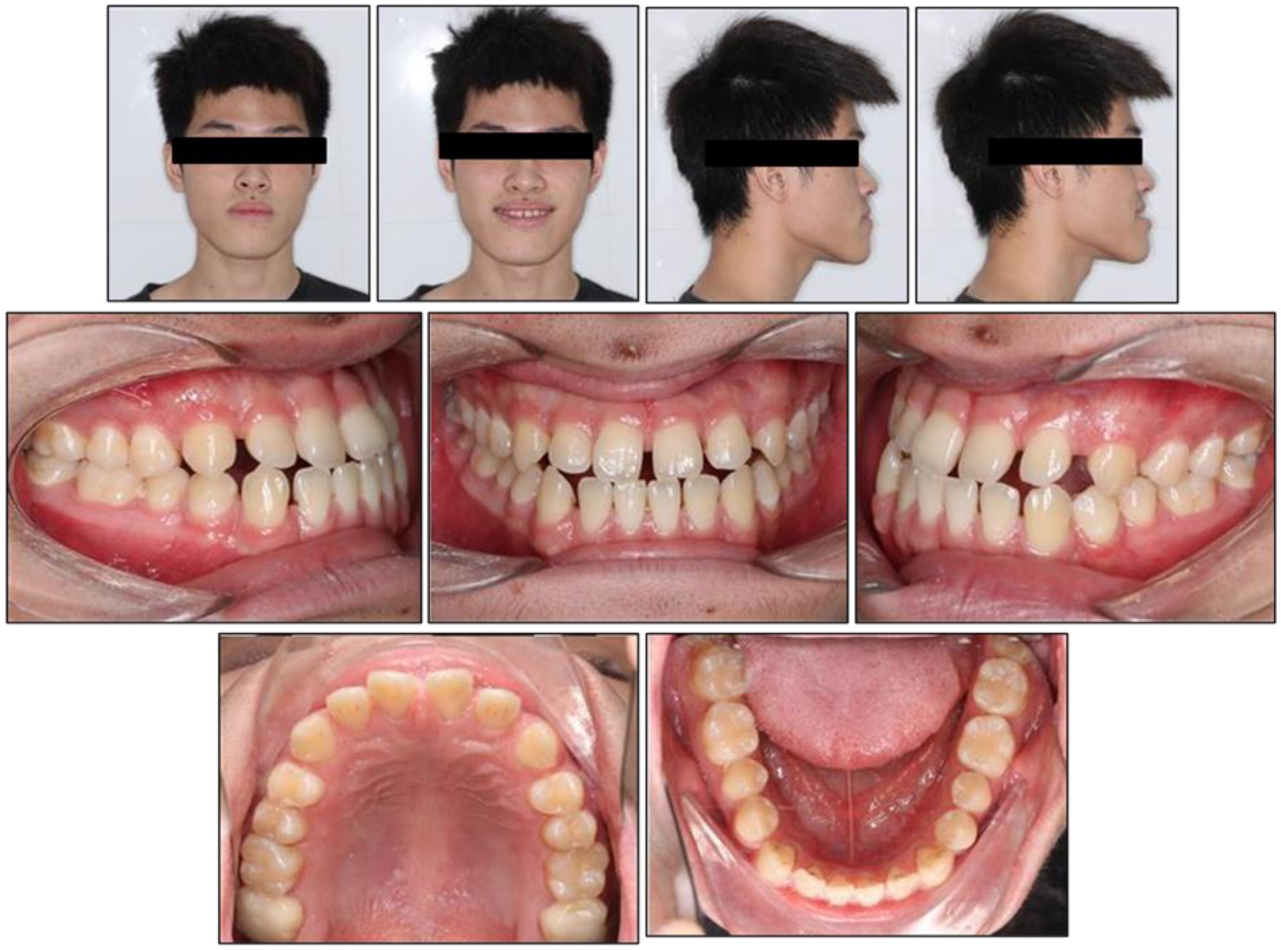
Pretreatment intraoral and extraoral photographs.

Extraorally (Fig. 1), frontal evaluation revealed a relatively long lower facial third with slight chin prominence and a rightward chin deviation. Lateral assessment revealed a V-shaped facial pattern with increased lower facial height and protrusion of the lower lip and chin. Upon smiling, approximately 80% of the maxillary incisors were displayed, and minimal buccal corridors were observed. There were no signs or symptoms of temporomandibular joint dysfunction, and functional excursions were within normal limits.

Intraorally, the patient exhibited bilateral class III canine and molar relationships, a 0-mm overjet and overbite, and generalized spacing – more pronounced in the maxilla than the mandible. The maxillary dental midline was shifted 1 mm to the right of the facial midline, while the mandibular dental midline was coincident.

**Figure 2.**
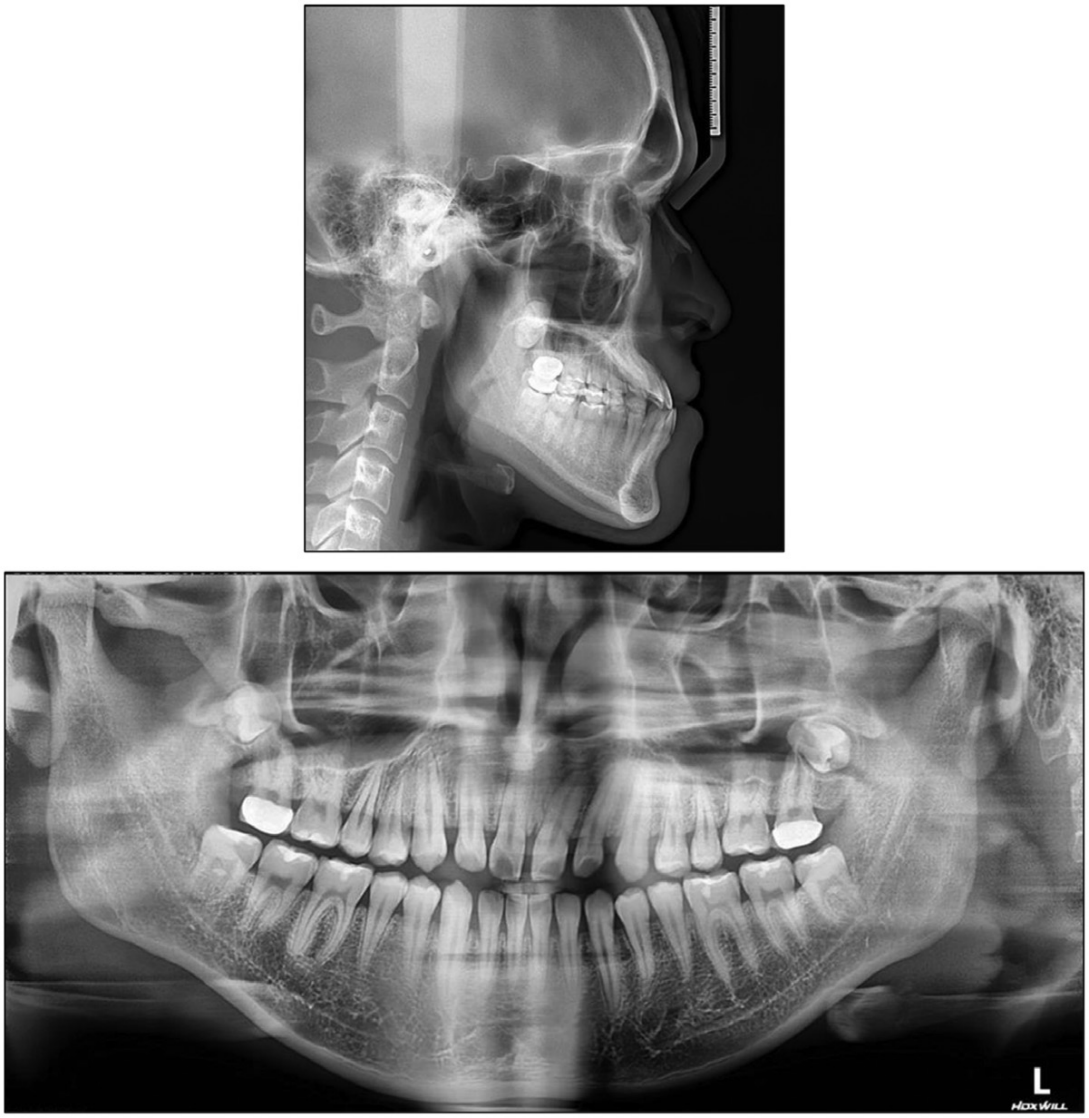
Pretreatment lateral cephalogram and panoramic radiograph.

The panoramic radiography (Fig. 2) showed the presence of all 4 third molars, full-coverage crowns on both maxillary second molars, and generalized horizontal and oblique alveolar bone loss around the dentition.

Pretreatment cephalometric (Table 1) analysis confirmed a marked skeletal class III sagittal discrepancy that was close to the surgical range, with a sella-nasion-A point angle of 85.81°, a sella-nasion-B point angle of 89.65°, an A point-nasion-B point angle (ANB) of −3.84°, and a Wits appraisal of −6.83 mm. Despite clinically increased lower facial height, the Frankfort-mandibular plane angle of 21.35° indicated mild hypodivergence. Dental compensation featured marked proclination of the maxillary incisors (upper incisor to sella-nasion angle 125.14°; upper incisor to nasion-A point angle and linear 41.41° and 12.19 mm) and slight lingual tipping of the mandibular incisors (incisor mandibular plane angle 86.61°; lower incisor to nasion-B point [L1-NB] angle and linear 24.57° and 5.94 mm), resulting in a −1.72 mm overjet and 1.29 mm overbite. Soft-tissue analysis showed retrusion of the upper lip (−5.62 mm) and normal position of the lower lip (0.34 mm) relative to Ricketts’ esthetic line (E-line).

**Table 1:** Cephalometric mesurements.

Mesurements	Norm (mean ± SD)	Pretreatment	Posttreatment
Skeletal			
SNA (°)	81.08 ± 3.7	85.81	85.55
SNB (°)	79.17 ± 3.8	89.65	88.43
ANB (°)	2.46 ± 1.8	−3.84	−2.88
FMA (°)	25 ± 4.0	21.35	21.99
Wits appraisal (mm)	−0.33 ± 2.7	−6.83	−2.15
Dental			
U1-SN (°)	109.31 ± 6.0	125.14	118.01
IPMA (°)	90 ± 3.5	84.78	75.62
U1-NA (°)	22.0 ± 5.0	39.33	32.46
U1-NA (mm)	4.0 ± 3.0	9.73	7.52
L1-NB (°)	25.0 ± 5.0	21.31	12.27
L1-NB (mm)	4.0 ± 2.0	5.77	1.34
Interincisal angle (°)	130.0 ± 5.0	123.20	138.15
Overjet (mm)	2.0 ± 2.0	−1.72	1.90
Overbite (mm)	2.0 ± 2.0	1.29	1.66
Tissue			
E-line-upper lip (mm)	0 ± 2.0	−5.62	−5.71
E-line-lower lip (mm)	0 ± 2.0	0.34	−3.98

ANB = A point-nasion-B point, E-line = esthetic line, FMA = Frankfort-mandibular plane angle, IMPA = incisor mandibular plane angle, L1-NB = lower incisor to nasion-B point, SNA = sella-nasion-A point, SNB = sella-nasion-B point, U1-NA = upper incisor to nasion-A point, U1-SN = upper incisor to sella-nasion.

Overall, the patient demonstrated a borderline skeletal class III malocclusion characterized by mandibular prognathism in a mildly hypodivergent growth pattern, a concave facial profile, bilateral angle class III canine and molar relationships, and an edge-to-edge incisor relationship, with generalized anterior spacing in both arches.

### 2.2. Treatment objectives

The treatment objectives were to camouflage the patient’s skeletal class III discrepancy by preserving the existing dentoalveolar compensation – maintaining the proclined maxillary incisors and lingually tipped mandibular incisors – while establishing bilateral angle class I canine and molar relationships. The anterior edge-to-edge incisor relationship was to be corrected to a functional overjet of 2 to 3 mm with a normal overbite, and all generalized spacing (particularly maxillary diastemas) was to be closed with alignment of the maxillary midline to the facial midline. Soft-tissue support was to be enhanced by improving lip posture relative to Ricketts’ esthetic line, with careful mechanics and periodontal monitoring to preserve the compromised alveolar bone levels. Finally, the goal was to achieve a stable, harmonious occlusion and smile esthetics maintained long-term through appropriate retention.

### 2.3. Treatment alternatives

Two management approaches were evaluated. First, orthognathic surgery – a mandibular setback via bilateral sagittal split osteotomy (with or without adjunctive maxillary procedures to optimize the occlusal plane) – was proposed in light of the marked skeletal discrepancy (Wits −6.83 mm) and near-limit dentoalveolar compensation.^[7,8]^ This option offers definitive correction of the class III skeletal relationship and predictable improvement of the concave soft-tissue profile, and a surgery-first sequence or limited presurgical decompensation may shorten overall treatment time.^[9]^ Its drawbacks include surgical invasiveness, higher cost, risk of inferior alveolar nerve neurosensory disturbance, and a potential reduction in pharyngeal airway dimensions, necessitating airway-conscious planning.^[10]^

Second, nonsurgical camouflage was considered by maintaining existing incisor compensations, extracting the mandibular third molars, and achieving en-masse mandibular total-arch distalization with TADs (buccal-shelf miniscrews) to obtain bilateral class I canine and molar relationships and a functional overjet under strict vertical control.^[3]^ Advantages include avoidance of surgery and premolar extractions, lower morbidity and cost, and reduced reliance on intermaxillary elastics; limitations are the lack of profile improvement, correction constrained by the mandibular posterior anatomic limit and periodontal support, and risks of root proximity or soft-tissue irritation around extra-alveolar devices.^[11]^

Given his satisfaction with his facial appearance and preference to avoid orthognathic surgery, the patient elected nonsurgical dentoalveolar camouflage consisting of en-masse mandibular total-arch distalization supported by TADs (buccal-shelf miniscrews).

### 2.4. Treatment progress

At appliance placement, 0.022 × 0.026-in-slot metal self-ligating brackets (Wepass SE; 3B, Hangzhou, China) with a McLaughlin-Bennett-Trevisi prescription were bonded in both arches, and alignment was initiated with 0.014-in nickel-titanium (NiTi) archwires. The lower incisor brackets (original torque −6°) were inverted to provide +6° torque, thereby preventing excessive lingual inclination during distalization. Shortly thereafter, 0.016-in NiTi archwires were placed and the mandibular third molars were extracted under local anesthesia; alignment progressed to 0.016 × 0.022-in NiTi and 0.017 × 0.025-in stainless steel (SS) with anterior consolidation.

**Figure 3.**
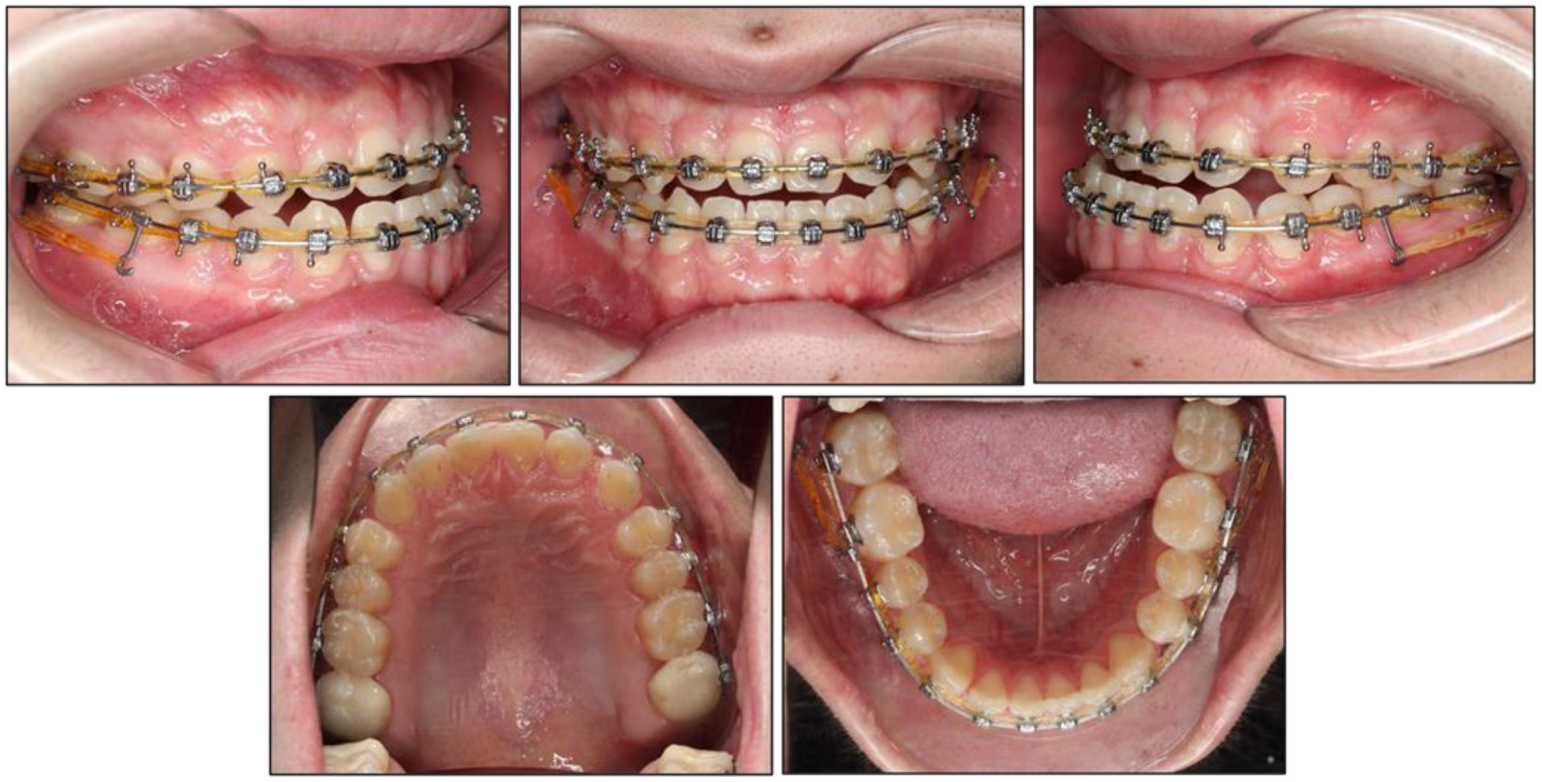
Intraoral photographs at 5 months into treatment.

After 5 months, partial decompensation of the incisor inclinations exposed the true anteroposterior disharmony, resulting in a more pronounced negative overjet (Fig. 3). Two 2.0 × 12-mm TADs (Hi-fix, Medico, Gyeonggi-do, Korea) were placed in the mandibular buccal shelf region, a 0.019 × 0.025-in SS archwire was engaged in the mandibular arch, and en-masse distalization was initiated with TAD-to-hook elastics while maxillary chains were refreshed. Subsequently, the mandibular posterior segment (from left to right first molars) was ligated as a unit to facilitate en-masse distalization, and short-term unilateral class III elastics were applied and discontinued once the sagittal goal was reached.

Thereafter, finishing included selected bracket repositioning; lingual auxiliaries were bonded to the mandibular anterior teeth for leveling lingual cusps and torque control with 0.016 × 0.016-in, then 0.017 × 0.025-in rectangular lingual wires. The maxillary arch progressed to 0.018 × 0.025-in SS and the mandibular labial arch to 0.019 × 0.025-in SS with added torque. Short settling and cross elastics were applied intermittently to refine transverse coordination and intercuspation.

**Figure 4.**
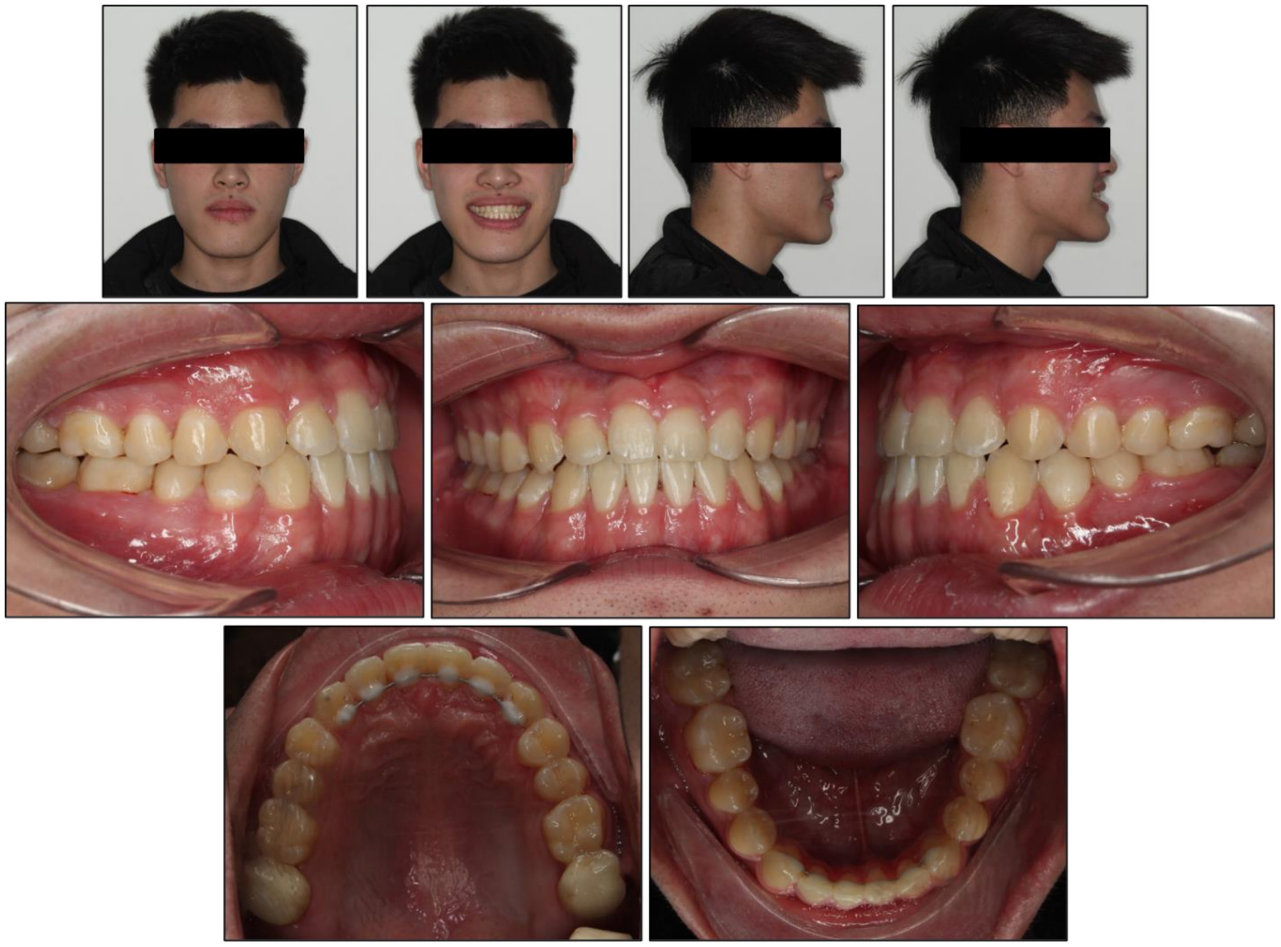
Posttreatment intraoral and extraoral photographs.

After approximately 15 months of active treatment (Fig. 4), appliances were removed. Fixed canine-to-canine lingual retainers were bonded in both arches, and clear vacuum-formed retainers were delivered for full-time wear initially.

### 2.5. Treatment results

Posttreatment (Fig. 4) extraoral records showed a more balanced facial appearance. Maxillary incisor display increased, the smile arc was fuller, and passive lip seal became easier. In profile, the contour improved from concave to nearly straight owing to enhanced upper-lip support and reduced lower-lip eversion; chin position and the lower facial third remained essentially unchanged.

Intraorally, bilateral angle class I canine and molar relationships were achieved. The arches were well aligned and coordinated, anterior spaces were closed, and the overjet and overbite were corrected to slight positive values (≈2 mm and ≈1 mm, respectively). The maxillary dental midline was corrected to coincide with the facial midline.

**Figure 5.**
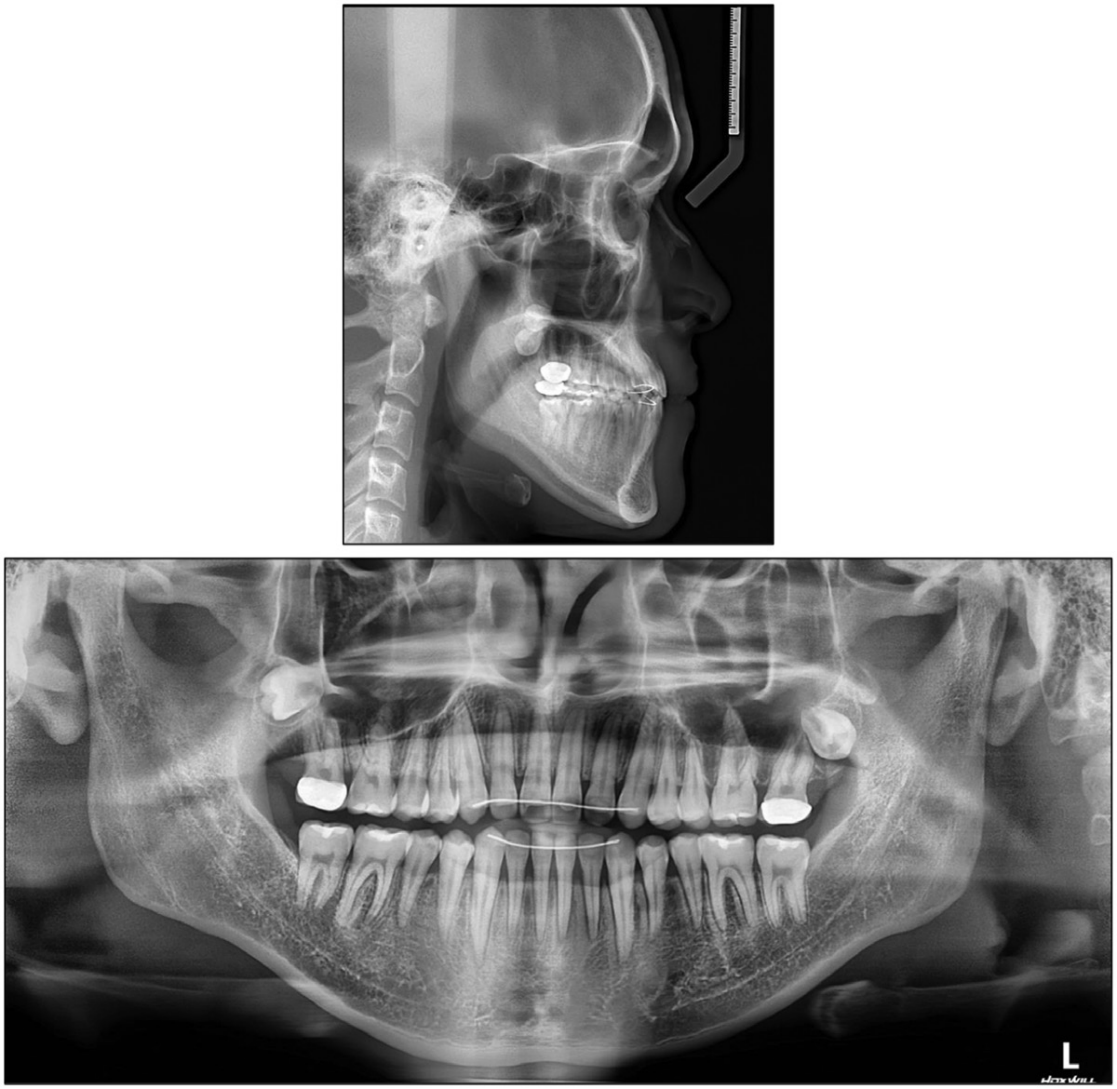
Posttreatment lateral cephalogram and panoramic radiograph.

The posttreatment panoramic radiograph (Fig. 5) showed parallel roots without evident external apical root resorption. Posttreatment cephalometric changes (Table 1) supported successful dentoalveolar camouflage driven by mandibular dentoalveolar distalization rather than true skeletal correction. Skeletal measurements of the maxilla remained stable (sella-nasion to A-point angle 85.81° to 85.45°), while the mandible was repositioned posteriorly (sella-nasion to B-point angle 89.65° to 88.43°). Mandibular plane angle showed a modest increase (Frankfort-mandibular plane angle 21.35° to 21.99°), which was favorable in this hypodivergent patient, inducing slight clockwise mandibular rotation and thereby further improving the anteroposterior relationship (ANB −3.84° to −2.88°). Accordingly, the Wits appraisal demonstrated substantial improvement (−6.83 mm to −2.15 mm). The mandibular dentition was distalized by 4.43 mm, indexed by the reduction in the lower incisor-to-nasion-B-point distance (L1-NB 5.77 to 1.34 mm), accompanied by moderate retroclination (incisor mandibular plane angle 84.78° to 75.62°; L1-NB angle 21.31° to 12.27°). Maxillary incisors were adequately uprighted and retracted (upper incisor to sella-nasion line angle 125.14° to 118.01°; upper incisor to nasion-A-point linear 9.73 to 7.52 mm) due to diastema closure. These movements converted the edge-to-edge bite to a positive overjet and overbite (1.90 and 1.66 mm). Clinically, the approximately 4-mm en-masse distalization represents a large magnitude of correction for adult class III camouflage.

## 3. Discussion

This case illustrates that, in a carefully selected borderline adult class III patient, buccal-shelf miniscrew–anchored mandibular dentoalveolar distalization can achieve clinically useful occlusal camouflage. The strategy avoided orthognathic surgery and premolar extractions, provided compliance-independent anchorage, and permitted simultaneous space closure and midline control.^[3]^ A large distal correction (approximately 4–5 mm) was feasible after removal of the mandibular third molars to create posterior clearance and was consistent with contemporary ranges for adult mandibular distalization.^[12]^ In the absence of cone-beam computed tomography (CBCT), planning relied on panoramic and lateral cephalometric records supplemented by clinical examination. Although 3-dimensional quantification of the mandibular posterior anatomic limit was not possible, posterior clearance was inferred after third-molar removal and monitored radiographically. Conservative biomechanics were used with strict vertical control and light continuous forces to minimize cortical contact, root proximity, and unwanted clockwise mandibular rotation.^[13]^ Under these safeguards, clinically meaningful distalization was achieved; the magnitude of correction was estimated indirectly from cephalometric changes, conversion from bilateral class III to class I intercuspation, and closure of anterior spaces, without evident external apical root resorption on the posttreatment panoramic radiograph.

Torque control was pivotal in this skeletal class III camouflage case. Because compensated mandibular incisors are typically retroclined, distalization and class III elastics without adequate torque control risk uncontrolled tipping with labial root displacement, jeopardizing periodontal support and yielding minimal skeletal effect. By actively expressing positive torque, lingual root movement can be encouraged, permitting alveolar remodeling around B-point. This may partly explain the improvement in ANB and Wits appraisal; however, these changes should not be interpreted as true basal skeletal correction. In this patient, lower incisor brackets were inverted to deliver positive torque, and a full-size 0.019 × 0.025-in stainless-steel main archwire was used with added torque.^[14]^ Additionally, distalization was achieved using buccal-shelf miniscrews combined with power arms to align the force vector closer to the center of resistance of the mandibular arch.^[2]^ This approach facilitated controlled tipping with lingual root movement. Collectively, the torque-focused biomechanics helped translate the mandibular incisors posteriorly without excessive retroclination, contributing to a limited but desirable B-point response and stable camouflage.

With respect to alternatives, orthognathic surgery (mandibular setback with or without maxillary procedures) remains indicated when skeletal disharmony and soft-tissue demands exceed the capacity of dentoalveolar movements; surgery-first sequencing may shorten overall treatment duration while maintaining early stability, but at the cost of surgical morbidity and airway-related considerations. In the present patient, the small initial negative overjet, mildly hypodivergent pattern (favorable for vertical control), generalized spacing, and third-molar removal collectively supported a nonsurgical camouflage approach with mandibular total-arch distalization.^[9,15]^ Reductions in oropharyngeal airway dimensions have been reported after isolated mandibular setback, underscoring the need for airway-conscious planning.^[16]^

Relative to the current literature, this borderline class III case achieved upper-range distalization of approximately 4 to 5 mm, whereas systematic reviews and cohort syntheses typically report ~3 to 5 mm with consistent occlusal improvements but predictably limited soft-tissue change compared with orthognathic surgery.^[3]^ Posttreatment stability in matched cohorts has been shown to be comparable to mandibular setback surgery, aligning with the clinical course observed here.^[5]^ Biomechanically, the protocol adopted is consistent with contemporary recommendations for total-arch distalization.^[13]^ Finally, because the mandibular posterior anatomic limit varies with facial pattern, hypodivergent patients generally afford greater posterior clearance – supporting the case selection here and the frequent recommendation to obtain CBCT when planning large distal movements.^[17]^

This single-patient case report lacks a control group, limiting generalizability and causal inference. Distalization was estimated indirectly from 2D cephalometric surrogates because CBCT was not obtained, so the mandibular posterior anatomic limit, buccolingual alveolar housing, root proximity to cortical boundaries, and true 3-dimensional magnitude of distalization could not be directly evaluated. Panoramic imaging may under-detect minor external apical root resorption and cannot assess transverse changes. Periodontal safety remains a limitation because generalized alveolar bone loss was evident on pretreatment panoramic radiography, but periodontal parameters were not systematically recorded before and after high-magnitude distalization and torque control.^[18]^ The posttreatment observation interval was short, precluding conclusions about long-term stability.

## 4. Conclusions

A carefully selected borderline adult skeletal class III patient who declined surgery was managed with buccal-shelf miniscrew–anchored en-masse mandibular dentoalveolar distalization after third-molar removal. The treatment achieved bilateral class I occlusion, space closure, and positive overjet and overbite. The correction should be interpreted primarily as dentoalveolar camouflage; favorable cephalometric changes likely reflected mandibular rotation and dentoalveolar changes rather than true skeletal correction.

## Author contributions

**Conceptualization:** Viet Anh Nguyen.

**Data curation:** Viet Anh Nguyen.

**Formal analysis:** Thi Minh Anh Ha.

**Investigation:** Thi Minh Anh Ha, Viet Hoang.

**Methodology:** Viet Anh Nguyen.

**Project administration:** Viet Anh Nguyen, Anand Marya.

**Resources:** Viet Anh Nguyen.

**Software:** Viet Anh Nguyen.

**Supervision:** Viet Anh Nguyen.

**Validation:** Viet Anh Nguyen.

**Visualization:** Viet Anh Nguyen, Thi Minh Anh Ha.

**Writing – original draft:** Thi Minh Anh Ha, Anand Marya.

**Writing – review & editing:** Viet Anh Nguyen, Viet Hoang, Anand Marya.

## References

[1] Alhammadi MS Halboub E Fayed MS Labib A El-Saaidi C. Global distribution of malocclusion traits: a systematic review. Dental Press J Orthod. 2018;23:40.e1–40.e10. 10.1590/2177-6709.23.6.40.e1-10.onl PMC6340198 30672991

[2] Nguyen VA Nguyen NA Doan HL Pham TH Doan BN. Management of anterior and posterior crossbites with lingual appliances and miniscrew-assisted rapid palatal expansion: a case report. Medicine (Baltimore). 2024;103:e40832. 39654233 10.1097/MD.0000000000040832 PMC11630943

[3] Setvaji NR Sundari S. Evaluation of treatment effects of en masse mandibular arch distalization using skeletal temporary anchorage devices: a systematic review. Cureus. 2024;16:e71171. 39525243 10.7759/cureus.71171 PMC11550868

[4] Kim B Shin J Hong C. The lower lip profile change during total distalization of the mandibular dentition. Open Dent J. 2023;17:e187421062212261.

[5] Kook YA Choi TH Park JH Kim SH Lee NK. Comparison of posttreatment stability after total mandibular arch distalization with mini-implants and mandibular setback surgery. Angle Orthod. 2024;94:159–67. 38195065 10.2319/062723-447.1 PMC10893925

[6] Park JH Emamy M Lee SH. Adult skeletal class III correction with camouflage orthodontic treatment. Am J Orthod Dentofacial Orthop. 2019;156:858–69. 31784020 10.1016/j.ajodo.2018.07.029

[7] Huang CS Hsu SS Chen YR. Systematic review of the surgery-first approach in orthognathic surgery. Biomed J. 2014;37:184–90. 25116713 10.4103/2319-4170.126863

[8] Benington P Anwar M Mohan A Gillgrass T Ayoub A. Outcome measures of the surgery first approach for orthognathic correction of dentofacial deformities. Br J Oral Maxillofac Surg. 2024;62:71–5. 38057176 10.1016/j.bjoms.2023.10.023

[9] Park YW Kwon KJ Kang YJ Jang IS. Surgery-first approach reduces the overall treatment time without damaging long-term stability in the skeletal class III correction: a preliminary study. Maxillofac Plast Reconstr Surg. 2021;43:27. 34273017 10.1186/s40902-021-00304-8 PMC8286210

[10] Roychoudhury S Nagori SA Roychoudhury A. Neurosensory disturbance after bilateral sagittal split osteotomy: a retrospective study. J Oral Biol Craniofac Res. 2015;5:65–8. 26258016 10.1016/j.jobcr.2015.04.006 PMC4523587

[11] Rustico L Ronsivalle V Iaculli F Spagnuolo G Isola G Giudice AL. Class III orthodontic camouflage: is the “ideal” treatment always the best option? A documented case report. Case Rep Dent. 2022;2022:9200469. 35865552 10.1155/2022/9200469 PMC9296280

[12] Inchingolo AM Patano A Malcangi G. Mandibular molar distalization in class III malocclusion: a systematic review. Appl Sci. 2023;13:9337.

[13] Park JH Heo S Tai K Kojima Y Kook Y-A Chae J-M. Biomechanical considerations for total distalization of the mandibular dentition in the treatment of class III malocclusion. Seminars Orthod. 2020;26:148–56.

[14] Son TM Anh NV Ninh DV. Multidisciplinary orthodontic-orthognathic management of severe skeletal class III malocclusion and anterior open bite. APOS Trends Orthod. 2025;15:188–94.

[15] Soverina D Gasparini G Pelo S. Skeletal stability in orthognathic surgery with the surgery first approach: a systematic review. Int J Oral Maxillofac Surg. 2019;48:930–40. 30685226 10.1016/j.ijom.2019.01.002

[16] Tan SK Leung WK Tang ATH Zwahlen RA. Effects of mandibular setback with or without maxillary advancement osteotomies on pharyngeal airways: an overview of systematic reviews. PLoS One. 2017;12:e0185951. 29016682 10.1371/journal.pone.0185951 PMC5633244

[17] Kim SH Cha KS Lee JW Lee SM. Mandibular skeletal posterior anatomic limit for molar distalization in patients with class III malocclusion with different vertical facial patterns. Korean J Orthod. 2021;51:250–9. 34275881 10.4041/kjod.2021.51.4.250 PMC8290085

[18] Rathee M Rao PL Bhoria M. Prevalence of gingival biotypes among young dentate North Indian population: a biometric approach. Int J Clin Pediatr Dent. 2016;9:104–8. 27365928 10.5005/jp-journals-10005-1343 PMC4921876

